# Mechanisms of Hearing Loss in Neurofibromatosis Type 2

**DOI:** 10.1371/journal.pone.0046132

**Published:** 2012-09-26

**Authors:** Ashok R. Asthagiri, Raul A. Vasquez, John A. Butman, Tianxia Wu, Keaton Morgan, Carmen C. Brewer, Kelly King, Chris Zalewski, H. Jeffrey Kim, Russell R. Lonser

**Affiliations:** 1 Surgical Neurology Branch, National Institute of Neurological Disorders and Stroke, National Institutes of Health, Bethesda, Maryland, United States of America; 2 Department of Neurological Surgery, University of Florida, Gainesville, Gainesville, Florida, United States of America; 3 Radiology and Imaging Sciences, The Clinical Center at the National Institutes of Health, National Institutes of Health, Bethesda, Maryland, United States of America; 4 Clinical Neurosciences Program, National Institute of Neurological Disorders and Stroke, National Institutes of Health, Bethesda, Maryland, United States of America; 5 Audiology Unit, Otolaryngology Branch, National Institute on Deafness and Other Communication Disorders, National Institutes of Health, Bethesda, Maryland, United States of America; 6 Office of the Clinical Director, National Institute on Deafness and Other Communication Disorders, National Institutes of Health, Bethesda, Maryland, United States of America; City of Hope, United States of America

## Abstract

**Introduction:**

Patients with neurofibromatosis type 2 (NF2) develop bilateral cochleovestibular schwannomas (CVSs) that cause binaural deafness in most individuals. Hearing loss occurs in an unpredictable manner and the underlying mechanisms are not known. To gain insight into the pathophysiologic basis for hearing loss in NF2, we performed a prospective cross-sectional study of untreated ears in NF2 patients.

**Methods:**

One hundred consecutive NF2 patients in a prospective natural history study were included. Clinical and audiometric data were analyzed for treatment naïve ears. In addition to standard MR-imaging sequences, alterations in intralabyrinthine protein content were determined utilizing high resolution FLAIR, the presence of cochlear aperture obstruction was determined by examining 3D T2 sequences, and endolymphatic hydrops was identified on delayed post-contrast FLAIR sequences.

**Results:**

Eighty-nine ears harboring 84 untreated CVSs in 56 consecutive NF2 patients (age 30±16 years) were analyzed. Thirty-four (38%) ears had varying degrees of hearing loss. Elevated intralabyrinthine protein was identified in 70 (75%) ears by FLAIR MR-imaging and was strongly associated with the presence of hearing loss (32/34 hearing loss ears; 94%)(Fisher's exact test; P = .005). Elevated intralabyrinthine protein was associated with the presence of CVS-associated cochlear aperture obstruction (64 of 67 ears with elevated protein; 96%)(Fisher's exact test; P<0.0001) in both normal and hearing loss ears. Elevated intralabyrinthine protein was not identified in ears without CVS (5 ears). While larger tumor size was associated with hearing loss (P = 0.006), 16 hearing loss ears (47%) harbored CVSs less than 0.5 cm^3^, including 14 ears (88%) with block of the cochlear aperture and elevated protein.

**Discussion:**

These findings are consistent with a model in which hearing loss develops as a result of cochlear aperture obstruction and accumulation of intralabyrinthine protein. MRI based identification of elevated intralabyrinthine protein may help identify the ear at-risk for developing hearing loss.

## Introduction

Neurofibromatosis type 2 (NF2) is an autosomal dominant heritable neoplasia syndrome. It results from a germline mutation of the *NF2* tumor suppressor gene located on the long arm of chromosome 22 [1]. NF2 has an incidence of 1 in 25,000 persons and a penetrance of nearly 100% by age 60 years [2], [3]. While the clinical manifestations of NF2 include central and peripheral nervous system tumors (nerve sheath tumors, meningiomas and ependymomas), cutaneous lesions, ocular pathology and peripheral neuropathy [4], the hallmark of NF2 is the development of bilateral cochleovestibular schwannomas (CVSs) [5]. Bilateral CVSs are present in 90 to 95% of NF2 patients [5]. Although CVSs are benign tumors, they cause significant audiovestibular morbidity, including deafness. While binaural hearing loss occurs in nearly all NF2 patients, its course remains unpredictable and therefore management paradigms have remained inconsistent.

Despite the significant audiologic morbidity identified in patients with NF2, the mechanisms by which the CVS causes hearing loss are not understood. The most frequently cited hypothesis is the enlarging CVS causes hearing loss through direct compression and stretching of the cochlear nerve. Several phenomena, including the unpredictable onset of hearing loss, frequent association of hearing loss with small tumors and progressive hearing loss in patients with non-growing CVS specifically suggest that such a simple association between tumor size and/or growth rate with hearing loss does not exist [6], [7], [8], [9]. Further, in addition to gradual hearing loss, progression of hearing loss may be stepwise, relapsing and remitting, or sudden. These variable patterns of hearing loss suggest that additional mechanisms of hearing loss such as intralabyrinthine hemorrhage, development of endolymphatic hydrops, disruption of cochlear vascular supply and alteration in the biochemical milieu of inner ear fluids that result in cochlear hair cell degeneration and dysfunction may play a more critical role in the pathophysiology of hearing loss in patients with NF2. Because of the incomplete understanding of the pathophysiologic mechanisms underlying onset and variable progression of hearing loss in NF2 (even between ears in the same patient), the optimal management of CVSs in NF2 has not been determined. To identify markers for hearing loss and underlying pathophysiological mechanisms, we prospectively analyzed the clinical, imaging, and audiologic findings in a large cohort of NF2 patients.

## Methods

### Patients

Patients enrolled in a National Institute of Neurological Disorders and Stroke (NINDS) institutional review board (IRB)-approved prospective NF2 natural history study (NIH#08-N-0044) were included. Informed written consent was obtained from all adult participants. Informed written consent from the next of kin, carers or guardians on behalf of minors participating in this study was obtained. Both written consent and assent forms were approved for the consenting procedure by the NINDS IRB. Treatment naïve ears with no evidence of conductive hearing loss were used for analysis. Patients had NF2 diagnosed by clinical and/or genetic criteria [10].

### Patient Evaluation

#### Clinical and audiologic evaluation

Detailed histories were obtained and comprehensive otologic and neurologic examinations were conducted in all patients to exclude the presence of middle ear disease. Concurrent audiometric evaluations were performed that included air- and bone- conduction thresholds from 250–8000 Hz and 250–4000 Hz, respectively. Hearing was stratified utilizing the four frequency pure-tone average (PTA) as normal [PTA≤20 dB], mild hearing loss [PTA 21–40 dB], moderate [PTA 41–70 dB], severe [PTA 71–95 dB], or profound [PTA>95 dB] [11]. _ Ears with a conductive hearing loss component (>10 dB difference between the air and bone PTA) were excluded from the study. For statistical analysis, hearing status was simplified to a binary score based on the presence (mild, moderate, severe, profound) or absence (normal) of hearing loss.

#### Imaging evaluation

Patients underwent magnetic resonance (MR)-imaging with and without contrast (T1 weighted) of the craniospinal axis. A neuroradiologist, blind to the hearing status of individual patients and ears, interpreted all imaging findings. Inner ear MR-imaging was performed with less than 1 mm in plane resolution utilizing a 3T-MR-scanner (Phillips; Andover, MA). Non-contrast enhanced fluid attenuated inversion recovery (FLAIR) sequences were performed to assess for the presence of elevated protein within the inner ear. Because saline-based solutions become hyperintense relative to brain parenchyma at defined protein concentration thresholds, identification of elevated intralabyrinthine protein by pre-contrast FLAIR MR-imaging is dependable and was reported as either being “elevated” or “normal” [12]. Co-registered T2-VISTA MR-images were performed to assess for the presence of cochlear aperture obstruction, as defined by the absence of a patent cerebrospinal fluid (CSF) pathway between the posterior fossa and the cochlear aperture (cochlear nerve entry point into the osseous spiral ligament). The presence or absence of intralabyrinthine hemorrhage was assessed on precontrast T1-weighted MR-images [13]. To assess for endolymphatic hydrops, delayed FLAIR and matching T2-weighted images were obtained in a plane parallel to that of the horizontal semicircular canal 6 to 18 hours after contrast injection MR-imaging [14], [15]. On the delayed FLAIR, marked distension of the scala media (normally not visible) due to the differential elimination of contrast from the endolymphatic compartment relative to the retention of contrast material within the perilymph was noted as direct visualization of endolymphatic hydrops.

Volume of the CVS was determined utilizing post-contrast T1-weighted images [16] and the following formula: volume = maximum anteroposterior dimension X maximal mediolateral dimension X maximum craniocaudal dimension / 2) [17]. Volumetric measurements for CVSs were divided into posterior fossa and canalicular components if portions of the tumor [16] were present in both anatomic areas. The multilobulated posterior fossa component of tumors was further divided into individual compartments to improve accuracy of volumetric measurement (compartmental volume determined by equation defined above). Total CVS volume was determined by summing the volumes of the individual compartments. Identification of small discrete intralabyrinthine tumors by post-contrast images was confirmed by the presence of a negative outline on T2 VISTA sequences in co-registered images.

### Statistical Analysis

Statistical analyses were performed as defined in text. A P-value less than or equal to 0.05 was considered significant.

## Results

### Patient Characteristics

Of 100 consecutive patients (200 ears) enrolled into the NF2 natural history study, ears that underwent surgery (84 ears in 58 patients) and/or received radiation therapy (26 ears in 20 patients) for management of cochleovestibular schwannomas were excluded from this study. Additionally, 5 patients (10 ears) receiving chemotherapeutics at the time of this study and 1 patient (2 ears) unable to complete audiometric examination (English second language) were also excluded from evaluation. Three ears with mild hearing loss were also excluded from analysis due to the presence of a conductive hearing loss component by otologic and audiologic evaluation. Therefore, 89 treatment naïve ears (43 right, 46 left) in 56 consecutive patients (17 male, 39 female) were included. In 23 patients (41%), one ear met criteria for inclusion and in 33 patients (59%) both ears met inclusion criteria. MR-imaging evidence of CVS was present in 84 of the 89 ears; 5 ears had no MR-imaging evidence of CVS. Mean patient age at evaluation was 30±16 years (range, 8 to 68 years). Forty-nine patients (88%) had audiovestibular symptoms associated with a CVS (Table 1). Mean age at onset of any disease related symptom was 21.7±14.5 years (range, 0.3 to 57 years).

**Table 1:** Audiovestibular and MR-imaging findings in the 56 study patients (89 ears).

Findings	Number of patients/ears (%)*
None	7 patients (12.5%)
Hearing loss	34 ears (38.2%)
Tinnitus	48 ears (53.9%)
Vertigo	8 patients (14.2%)
Aural fullness	9 ears (10.1%)

MR-imaging characteristic	Normal Hearing ears	Hearing Loss ears	Total (n = 89)
Elevated protein	35	32	67 (75%)
Cochlear aperture block	36	31	67 (75%)
Intralabyrinthine tumor	5	8	13 (14.6%)
*Discrete*	*2*	*3*	*5 (5.6%)*
*Direct invasion*	*3*	*5*	*8 (9.0%)*
Endolymphatic hydrops	4	3	7 (7.9%)
Intralabyrinthine hemorrhage	0	0	0
Brainstem stroke	0	0	0

* Specific to the ear analyzed in the current study.

### Audiologic Findings

Fifty-five ears (62% of all evaluated ears) had normal hearing (PTA≤20 dB), including 5 (6%) ears without a CVS. Thirty-four ears (38%) had associated hearing loss (mild, 14 ears, 16%; moderate, 12 ears, 13%; severe, 4 ears, 4%; profound, 4 ears, 4%). Mean PTA was 54±29 dB (range, 22.5 to 118.75 dB) in the ears with associated hearing loss. Hearing loss occurred gradually over months to years (12 ears; 35% of ears with hearing loss), in a stepwise manner characterized by periodic decrements in hearing associated with intervening periods of stable hearing (10; 29%), in a relapsing-remitting pattern defined by periodic decrements in hearing coupled with partial recovery of hearing during intervening periods (4; 12%), or in a sudden and complete fashion (sudden profound sensorineural hearing loss) (5; 15%). Subjective hearing loss was not reported in 3 ears (9%) in 3 patients with audiometric evidence of mild hearing loss.

### Imaging Correlates in Hearing Loss Ears

#### Imaging and audiologic findings

**Figure 1:**
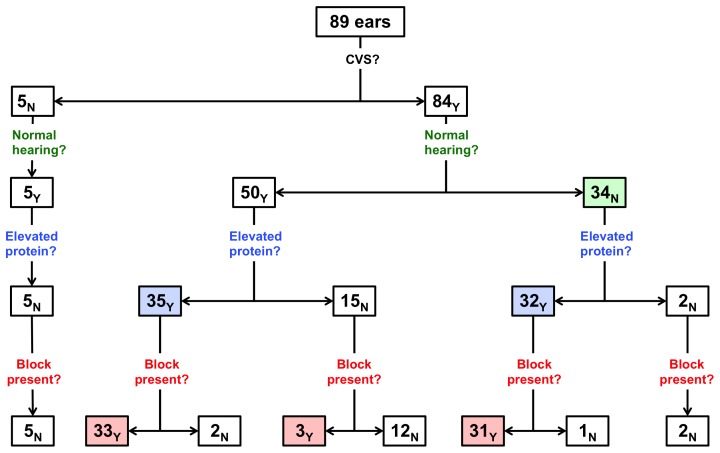
Features of normal hearing and hearing loss ears.

Radiographic and audiologic analysis in the 34 hearing loss ears revealed that 32 ears (94% of ears with hearing loss) had elevated intralabyrinthine protein on FLAIR MR-imaging (Figure 1). The 2 hearing loss ears without elevated intralabyrinthine protein had auditory brainstem responses that suggested central nervous system dysfunction or were associated with a large CVS (greater than 2.5 cm^3^). Other imaging findings and audiovestibular symptoms in the 56 study patients (89 ears) are detailed in Table 1.

#### Mechanism of elevated intralabyrinthine protein

**Figure 2:**
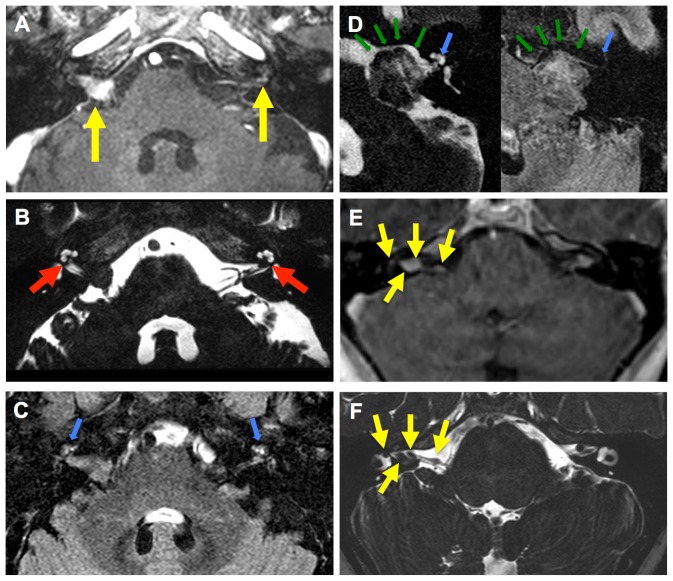
Magnetic resonance imaging in patients with neurofibromatosis type 2. Magnetic resonance (MR) imaging performed in a 47-year old patient with neurofibromatosis type 2 (NF2): (A) Axial, contrast enhanced, T1-weighted, magnetic resonance imaging demonstrates bilateral cochleovestibular schwannomas (CVS) (yellow arrows). (B) T2-VISTA sequence identifies the presence of a cerebrospinal fluid obstruction between the posterior fossa and the cochlear aperture (red arrows) bilaterally, independent of tumor size. (C) Pre-contrast fluid attenuated inversion recovery (FLAIR) sequence demonstrates the presence of elevated protein within the labyrinth (blue arrows). MR-imaging performed in a 48-year old patient with NF2: (D) Axial T2-VISTA and pre-contrast FLAIR sequences demonstrate the presence of normal labyrinthine protein (blue arrow) in the setting of a large CVS with an open CSF pathway (green arrows) between the posterior fossa and the cochlear aperture. MR-imaging performed in a 8-year old patient with NF2. (E) Axial, contrast enhanced, T1-weighted, magnetic resonance imaging demonstrates bilateral cochleovestibular schwannomas (CVS) with multiple tumors present on the right (yellow arrows). (F) T2-VISTA sequence identifies 4 discrete tumors, including a discrete intralabyrinthine tumor (yellow arrows).

Cochlear aperture obstruction by a CVS was closely associated with identification of elevated intralabyrinthine protein in hearing loss ears (Fisher's exact test; P = 0.005). While 31 hearing loss ears (91%) with elevated protein in the labyrinth had cochlear aperture obstruction by a CVS, only 1 ear (3%) with elevated intralabyrinthine protein did not have evidence of cochlear aperture obstruction (Figure 1, 2). Ears with hearing loss without elevated intralabyrinthine protein (2 ears, 6%) did not demonstrate cochlear aperture obstruction.

Twenty-three ears (out of 31 hearing loss ears with elevated protein and cochlear aperture obstruction; 74%) had isolated cochlear aperture obstruction and 8 (26%) had cochlear aperture obstruction associated with either a discrete intralabyrinthine tumor (3 ears) (Figure 2) or direct invasion of the labyrinth by a CVS (5 ears). Three ears with elevated intralabyrinthine protein, cochlear aperture obstruction and discrete intralabyrinthine tumors (1 ear) or direct invasion of the labyrinth by a CVS (2 ears) also had endolymphatic hydrops based on MRI findings.

### Imaging Correlates in Normal Hearing Ears

#### Imaging findings

Fifty normal hearing ears (91%) harbored a CVS and 5 ears (9%) did not have an associated CVS. Radiographic and audiologic analysis of the 50 normal hearing ears with a CVS demonstrated elevated intralabyrinthine protein in 35 ears (70% of all normal hearing ears with a CVS) and normal intralabyrinthine protein in 15 ears (30%) on FLAIR MR-imaging. The 5 normal hearing ears without CVS did not have evidence of elevated intralabyrinthine protein (Figure 1).

#### Mechanism of elevated intralabyrinthine protein

Similar to hearing loss ears, cochlear aperture obstruction by a CVS was associated with elevated intralabyrinthine protein in normal hearing ears (Fisher's exact test; P<0.0001). While 33 normal hearing ears (94% of normal hearing ears with elevated intralabyrinthine protein) with elevated intralabyrinthine protein had cochlear aperture obstruction by a CVS, only 2 ears (6%) with elevated intralabyrinthine protein did not have evidence of cochlear aperture obstruction.

Specific factors associated with elevated intralabyrinthine protein included isolated cochlear aperture obstruction by CVS (28 ears; 80%), cochlear aperture obstruction with direct labyrinthine invasion by CVS (3 ears; 9%), cochlear aperture obstruction with a discrete intralabyrinthine tumor (2 ears; 6%) or unknown mechanism (2 ears, 6%). Endolymphatic hydrops was also detected by MR-imaging in 3 normal hearing ears with elevated protein and block of the cochlear aperture.

Twelve normal hearing ears harboring a CVS (80% of all normal hearing ears without elevated labyrinthine protein) without elevated intralabyrinthine protein did not have block of the cochlear aperture, including 1 ear (8%) with associated hydrops. Three normal hearing ears (20%) had evidence of cochlear aperture block by CVS without elevated protein.

### Tumor Volume and Hearing Loss

**Figure 3:**
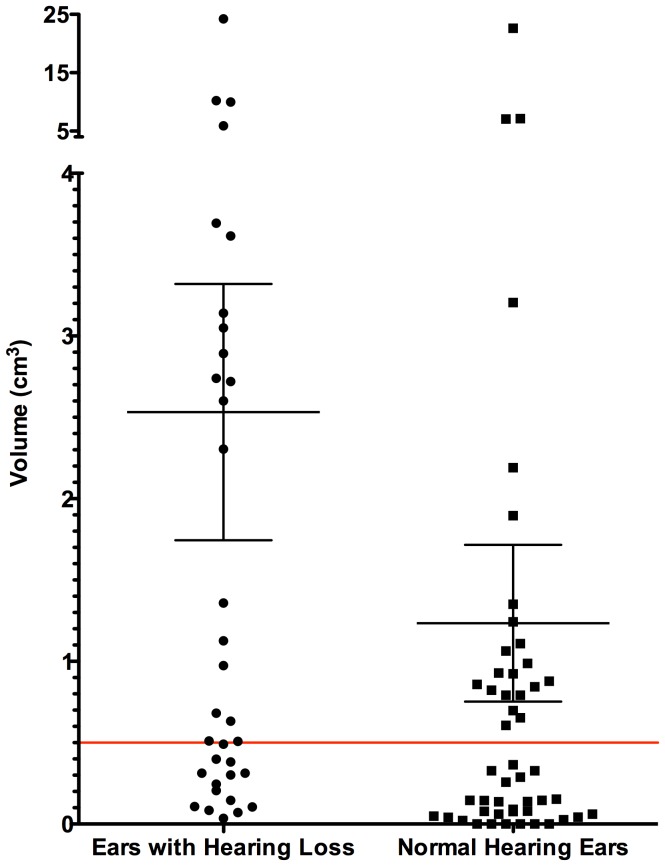
Graph demonstrating volume of tumors identified in ears with and without evidence of hearing loss.

CVSs were identified in 84 ears (94%) and were purely intracanalicular in 38 ears (45%). Mean tumor volume (in 84 ears) was 1.8±4.0 cm^3^ (approximately 1.5 cm in maximum linear dimension). While the presence of hearing loss was correlated with larger CVS volume in hearing loss ears (mean volume in normal hearing ears, 1.2±3.4 cm^3^ versus hearing-loss ears, 2.5±4.6 cm^3^)(Mann-Whitney; P = 0.007), 16 (47%) hearing loss ears had a CVS that was less than approximately 0.5 cm^3^ (Figure 3). Among 38 ears with a purely intracanalicular CVS, hearing loss was evident in 12 ears (32%) with a mean volume of 0.2±0.2 cm^3^ (range, 0.03 cm^3^ to 0.7 cm^3^).

## Discussion

### NF2-Associated Hearing Loss

Previous studies have demonstrated that CVS-associated binaural hearing loss will occur in an unpredictable manner over the lifetime of most NF2 patients [6], [7], [8]. Moreover, hearing loss patterns in one ear do not predict the clinical course of the contralateral ear in the same individual with NF2 [7]. Because of the uncertain clinical course associated with CVSs and because of the lack of understanding of the mechanisms that underlie hearing loss associated with CVSs in NF2, the management of these tumors has not been consistent or optimized. Here, we examined radiographic and audiometric findings associated with hearing loss in NF2 to better understand the pathophysiologic mechanisms underlying onset and progression of hearing loss in this hereditary neoplastic disorder.

### Current Study

#### Elevated intralabyrinthine protein

The most common imaging findings identified in CVS-associated hearing loss in NF2 was the presence of elevated intralabyrinthine perilymphatic protein and the presence of cochlear aperture obstruction on MR-imaging. Elevated intralabyrinthine protein was found in 94% of hearing loss ears (32 of 34 ears) and in 70% of normal hearing ears (35 of 50 ears). Blockage of the cochlear aperture by an associated CVS was found in 96% of ears (64 of 67 ears) with elevated protein and only in 14% of ears (3 of 22 ears) without evidence of elevated intralabrynthine protein. These findings are consistent with the findings by Silverstein and colleagues [18] that demonstrated that a 2.5-fold increase in intralabyrinthine protein obtained from perilymph aspirates could accurately identify the presence of CVSs in patients in the era before MR-imaging was available for non-invasive diagnosis.

The importance that elevated intralabyrinthine protein plays in hearing loss is underscored by previous clinical-imaging studies of the inner ear in other conditions, including sporadic vestibular schwannomas and sudden sensorineural hearing loss (SNHL) [19], [20]. Recent findings indicate that increased protein within the perilymph visualized on FLAIR MR-imaging can be correlated with hearing loss associated with sporadic acoustic schwannomas [20]. Elevated intralabyrinthine protein on FLAIR MR-imaging in patients with SNHL has also been correlated with hearing prognosis and extent of hearing loss that will persist [19].

#### Mechanisms of intralabyrinthine protein elevation

The findings of the current study indicate that cochlear aperture block underlies elevation of intralabyrinthine protein. The sensitivity and specificity of cochlear aperture obstruction in identifying elevated perilymphatic protein within the labyrinth was 96% and 87%, respectively. This indicates a pathophysiologic association between cochlear aperture obstruction and the presence of elevated intralabyrinthine protein may exist in NF2-associated CVSs. Based on the results from this study and previous studies examining elevated intralabyrinthine protein in other hearing loss syndromes, NF2-associated tumors may lead to elevated intralabyrinthine protein levels in several ways related to cochlear aperture obstruction that causes abnormal protein clearance and deposition.

Elevated intralabyrinthine protein could be the result of cochlear aperture obstruction and disruption of the blood-CSF/labyrinthine barrier caused by permeable tumor vessels [20]. CVSs cause increased protein content within the circulating cerebrospinal fluid compartment due to permeable tumor vessels, and this may be further concentrated in the lateral internal auditory canal in cases where CSF is trapped due to cochlear aperture obstruction [21], [22]. Concentrated proteins can rapidly diffuse into the perilymphatic space across the porous canaliculi located within the modiolar region of the osseous spiral lamina [23]. Previous studies have shown gadolinium accumulation in the perilymphatic compartment within 10 minutes after intravenous administration in ears with CVS compared to contralateral unaffected ears, supporting the presence of a breach in the blood-CSF/labyrinthine barrier and rapid diffusion into the perilymphatic spaces in ears with CVSs [20].

Another mechanism by which protein can accumulate within the perilymph associated with cochlear aperture obstruction involves impaired clearance caused by the CVS. It has been shown that the cochlear nerve is integral in the rapid transport of proteins from the cochlea to the cochlear nucleus [24]. Although large CVSs may impact the cochlear nerve through compression within the internal auditory canal disrupting axonal transport, small tumors situated within the cochlear nerve itself may also lead to cochlear aperture obstruction and disruption of axonal transport. Therefore, when CVSs cause obstruction of the cochlear aperture, independent of size, it is likely that this results in impaired cochlear nerve axonal transport that can, in turn, underlie perilymphatic protein accumulation due to impaired clearance [25].

Elevated intralabyrinthine protein could also be the result of increased protein production or deposition. Analogous to cerebellopontine angle CVSs that are known to cause increased protein content within the circulating cerebrospinal fluid compartment [21], increased protein secretion of a plasma ultrafiltrate by permeable tumor vessels of tumors within the labyrinth (both distinct and due to CVS invasion) may permit direct extravasation of plasma proteins into the inner ear perilymph. This process could potentially underlie (in part) the protein elevation and endolymphatic hydrops seen with these tumors. Similarly, increased tumor vessel permeability with leakage of plasma ultrafiltrate and plasma proteins into the surrounding anatomic region has been described in hemangioblastomas and endolymphatic sac tumors in von Hippel-Lindau disease and may underlie peritumoral cyst formation and inner ear hydrops [26].

#### Mechanisms of hearing loss associated with elevated protein

Elevated intralabyrinthine protein on MR-imaging was closely associated with the presence of hearing loss. The importance of elevated intralabyrinthine protein underlying hearing loss is supported by previous studies examining unique perilymphatic proteins that are elevated in CVSs, including μ-Crystallin (CRYM) and low density lipoprotein-related protein 2 (LRP2) [27]. Mutations of these proteins are associated with genetic syndromes that present with deafness not related to tumors. Further, previous studies have utilized histopathological evaluation to indirectly support biochemical degradation of the cochlea is caused by increased protein [28], [29], [30], [31]. Finally, multiple studies have revealed that hearing loss may progress in the absence of CVS growth [32], [33], confirming that an unknown factor underlies hearing loss in these ears. Although causality cannot be directly determined by the intimate association between elevated intralabyrinthine protein and hearing loss observed in our cohort, the high sensitivity of elevated protein in identifying hearing loss ears (94%) and the high negative predictive value of normal protein content in identifying a normal hearing ear (91%) presented in this study coupled with previous studies delineating the functional and degenerative sequelae of unique perilymphatic proteins that are elevated in the setting of vestibular schwannoma indicate that the increased protein content evident by labyrinthine FLAIR MR-signal may not only be an imaging marker of hearing loss, but contribute to the pathophysiology of hearing loss and cochlear degeneration.

#### Tumor size and hearing loss

A simple association between tumor size and hearing loss does not explain several phenomena. For instance, hearing loss in NF2 has an unpredictable onset and variable progression, which may be gradual, stepwise, relapsing and remitting, or sudden and complete. While previous studies have attempted to link increasing tumor size with hearing loss in patients with NF2, this association has not been established because of the ubiquitous presence of small tumors within all series that are associated with hearing loss. Because hearing loss in NF2 may be caused by end organ degeneration [34] (cochlea) from accumulated protein, the functional consequences of tumor location as it relates to the cochlear aperture appears to play an important role in the pathogenesis of hearing loss. This may also explain why schwannomas of various sizes in other cranial and peripheral nerves are often not associated with neurologic deficit in patients with NF2.

#### Alternative mechanisms of hearing loss in NF2

Other pathophysiologic processes of the inner ear structures have been implicated with hearing loss, including the development of endolymphatic hydrops and intralabyrinthine hemorrhage [13]. Although endolymphatic hydrops was identified in 7 ears (7.6%) with CVS in this study, the MR-based identification of endolymphatic hydrops did not correlate with hearing status (Table 1). Furthermore, the infrequent identification of endolymphatic hydrops and intralabyrinthine hemorrhage (0 ears), suggests these processes are not a significant underlying mechanism of hearing loss in this patient population.

#### Bevacizumab induced hearing response

Bevacizumab, initially utilized in NF2 clinical trials for the treatment of growing vestibular schwannomas was associated with hearing improvement in a subset of patients (4/7 patients, 57%) [35]. While this improvement in hearing was initially thought to be due to a reduction in intraneural edema and tumor shrinkage, a subsequent larger scale follow-up study confirmed a lack of correlation between radiographic response (tumor shrinkage) and hearing response, suggesting that a stochastic mechanism underlies the rapid hearing improvement (usually within 3 months) that is observed in approximately 50% of patients [36]. Because vascular endothelial growth factor (VEGF) is a known mediator of vessel permeability, bevacizumab (a VEGF-neutralizing antibody) may be affecting hearing response through restoration of protein homeostasis within the inner ear by decreasing production of protein rich plasma ultrafiltrate from both CPA and intralabyrinthine tumors.

#### Identification of the at-risk ear

The appropriate stratification of benefit to risk ratio for hearing preservation attempts in NF2 requires delineation of at-risk ears for hearing loss. Because hearing loss is currently the most likely outcome in NF2 ears, it becomes imperative to avoid risk when hearing loss is unlikely to occur. Despite the variable presentation of hearing loss in NF2, most ears with normal perilymphatic protein signal had normal hearing (20/22 ears, 91%) and most ears with hearing loss had elevated perilymphatic protein (35/37 ears, 95%).

Based on the available body of evidence and the current findings that indicate elevated perilymphatic proteins may underlie the final common pathway leading to hearing loss, MR-identification of labyrinthine hyperintensity in normal hearing ears (35 of 50 ears in this study) may serve as a potent imaging biomarker of ears at-risk for developing hearing loss.
